# Study on the liver Drug’s dominant metabolic enzymes for six effective components of the Huang qi Liuyi decoction

**DOI:** 10.3389/fphar.2023.1175896

**Published:** 2023-04-14

**Authors:** Qun Wang, Tiantian Tang, Zengguang Wu, Hong Yang, Yuan Gao, Shiyu Zhang, Xinli Song, Xiaolan Chen

**Affiliations:** ^1^ Guizhou University of Traditional Chinese Medicine, Huaxi University Town, Guiyang, China; ^2^ National Research Center of Miao Medicine and Engineering Technology, Huaxi University Town, Guiyang, China

**Keywords:** Huangqi Liuyi decoction, six effective components, CYP450 enzymes, liver microsomal, liver microsomal incubation *in vitro*

## Abstract

**Objective:** To investigate the dominant metabolic enzymes of six effective components (astragaloside IV, glycyrrhizic acid, calycosin-glucuronide, formononetin, ononin, calycosin-7-O-β-D- glucoside) of Huangqi Liuyi decoction extract (HQD).

**Methods:** Mouse liver microsomes were prepared. The effects of specific inhibitors of CYP450 enzymes on the metabolism of six effective components of HQD were studied using liver microsomal incubation *in vitro*.

**Results:** The chemical inhibitors of CYP2C37 inhibit the metabolism of glycyrrhizic acid and astragaloside IV. Formononetin and astragaloside IV metabolism is inhibited by the chemical inhibitors of CYP2C11. The chemical inhibitors of CYP2E1 and CYP1A2 inhibit the metabolism of calycosin-glucuronide. Chemical CYP3A11 inhibitors prevent formononetin and glycyrrhizic acid from being metabolized. However, no inhibitor significantly affected the metabolism of ononin and calycosin-7-O-β-D-glucoside.

**Conclusion:** CYP2C37 may be involved in the metabolism of astragaloside IV and glycyrrhizic acid, the metabolism of astragaloside IV and formononetin may be related to CYP2C11, the metabolism of calycosin-glucuronide may be related to CYP1A2 and CYP2E1, and CYP3A11 may be involved in the metabolism of glycyrrhizic acid and formononetin. This research provides an experimental basis for exploring the pharmacokinetic differences caused by metabolic enzymes.

## 1 Introduction

Diabetic nephropathy (DN) is one of the most serious microvascular complications of diabetes mellitus (DM). The clinical manifestations are persistent proteinuria, edema, decreased renal function, hypertension. The majority of cases progress to end-stage renal failure. Early treatment of diabetic nephropathy is significant in improving the quality of life and survival rate (Liu et al., 2022; Liu et al., 2022). Huangqi Liuyi decoction, first published in the “Taiping Huimin and Pharmacy Bureau Fang,” is composed of 60 g of *Astragali* and 10 g of *Glycyrrhizae*, and has the effect of greatly tonifying lung qi, nourishing kidney water, and harmonizing the spleen and stomach. Huangqi Liuyi decoction has been shown to prevent islet damage, lower blood glucose and glycated hemoglobin in diabetic model mice. In addition, *Astragali* prevents the establishment of renal interstitial fibrosis and *Glycyrrhizae* can prevent diabetic nephropathy by lowering lipid, lowering glucose levels, and preventing anti-oxidative stress in the kidneys (Chen et al., 2014; Hou et al., 2017; Wen et al., 2018; Ma et al., 2019; Dai et al., 2022). Traditional Chinese medicine (TCM) involves a multi-component and multi-target approach. Therefore, the quality and preparation of TCM aren’t easy to control, which hinders the in-depth research of TCM and the development of new drugs. To improve the pharmacological properties, individual ingredients from a compounded prescription are isolated and formulated according with the compatibility theory of TCM. This allows for clear pharmacological effects, relatively stable nature, and can effectively compensate for the shortcomings of traditional prescriptions whose quality is not easily controlled (Wang and Liang, 2004; Hu et al., 2016; Chen et al., 2019). Previously, our team discovered that the combination of four components extracted from Huangqi Liuyi Decoction, namely, astragalus saponin, glycyrrhizic acid, astragalus flavone, and astragalus polysaccharide (AKA HQD), improved renal fibrosis and slowed the progression of diabetic nephropathy in db/db mice (Wang et al., 2022). The study also discovered no statistical difference between the efficacy of Huangqi Liuyi decoction and HQD in mice with diabetic nephropathy (Wang et al., 2022b). According to pharmacokinetic studies, mice with diabetic nephropathy exhibited greater absorption and delayed elimination of the six effective components (astragaloside IV, glycyrrhizic acid, calycosin-glucuronide, formononetin, ononin, calycosin-7-O-β-D-glucoside) following oral administration HQD (Wang et al., 2022c).

The pathological state of the patient considerably influences drug-related processes *in vivo*, which directly relates to the drug’s efficacy and side effects (Ma et al., 2021; Qian et al., 2022). The alterations in the pharmacokinetics of drug in DN may relate to the changes in the activity of a variety of CYP450 cytochrome (CYP) enzymes involved in drug absorption, metabolism, and excretion (Hu et al., 2014; Gong et al., 2015). In our preliminary research, DN induced the activity of CYP2C11 and CYP3A11. HQD inhibited the activity of CYP1A2, CYP2C37, and CYP3A11 and induced the activity of CYP2C11. Moreover, HQD induced the activity of CYP2E1 in the DN mouse and inhibited the activity of CYP2E1 in the control mouse. The activity of each CYP450 enzyme was consistent with changes in expression (Wang et al., 2022d). However, the specificity of individual CYP enzymes allows for only specific substrates to be metabolized by the enzyme. The enzyme primarily involved in the metabolism of a certain substrate is known as the dominant metabolizing enzyme. Analyzing the dominant metabolizing enzyme of a drug is essential for studying how variations in the activity and expression of the metabolizing enzyme effect the drug pharmacokinetics and to elucidate the pharmacokinetic differences in pathophysiological states. In the present research, an *in vitro* liver microsomal incubation method was adopted to study the influence of specific inhibitors of liver CYP450 enzymes on the metabolism of the six effective components (astragaloside IV, glycyrrhizic acid, calycosin-glucuronide, formononetin, ononin, calycosin-7-O-β-D- glucoside) of HQD so as to analyze the CYP enzymes that may be involved in the metabolism of each component. From the perspective of drug metabolism, we investigated the reasons for the pharmacokinetic differences of HQD during the pathological and physiological changes and the possible drug-drug interactions in clinical application. The information will help provide a theoretical basis for developing new drugs against diabetic nephropathy and the rationale for their clinical application.

## 2 Materials and methods

### 2.1 Materials and reagents

The China Institute of Food and Drug Control provided the following reference standards: astragaloside IV, glycyrrhizic acid, calycosin-glucuronide, formononetin, ononin, calycosin-7-O-β-D- glucoside, puerarin, and digoxin (purity >98.0%). Ticlopidine (693228-63-6), α-naiflavone (604-59-1), quinidine (56-54-2), fluconazole (86386-73-4), ketoconazole (65277-42-1), and clomethiazole (533-45-9) were supplied by Shanghai yuanye Bio-Technology Co., Ltd. (Shanghai, China). Nicotinamide adenine dinucleotide phosphate (NADPH, 704S082) and bicinchoninic acid protein assay kit (BCA, 20191123) were provided by Beijing Solarbio Science and Technology Co., Ltd. (Beijing, China). Merck KGaA (Germany) provided methanol and acetonitrile (HPLC grade). All remaining chemicals were of analytical grade. HQD was self-produced by the research group (Wang et al., 2022). The content of each component in HQD is shown in Table 1.

**TABLE 1:** Content of each component extracted from three batches of *Astragalus* and *Glycyrrhizae*.

Extraction fraction	Components	Content (%)
Extract of astragalus saponins	Astragalus saponins	72.17
	Astragaloside IV	2.72
Extract of glycyrrhizic acid	Glycyrrhizic acid	80.22
Extract of astragalus flavones	Astragalus flavones	71.58
	Calycosin-glucuronide	1.36
	Formononetin	0.33
	Calycosin-7-O-β-D-glucoside	1.71
	Ononin	0.86
Extract of astragalus polysaccharides	Astragalus polysaccharides	65.51

### 2.2 Animals

Ten-week-old db/db male mice (45 ± 5 g) and db/m mice (20 ± 2 g) were purchased from the Model Animal Research Center of Nanjing University and MOE Key Laboratory of Model Animal for Disease Study (SCXK [Su]2021–0016) and housed in polypropylene cages at a relative humidity of 60% ± 5%, constant temperature (25°C ± 1°C) and under a 12 h light/12 h dark cycle, mice were given free access to water and food. Twelve-week-old db/db mice developed nephropathy (Ponchiardi et al., 2013; Wang et al., 2022b). The animal studies were approved by the Animal Ethical Committee of Guizhou University of Traditional Chinese Medicine (the approval number is NO1702060).

### 2.3 Preparation of standard solutions

The following standard solutions were prepared in methanol. The concentrations of astragaloside IV, glycyrrhizic acid, calycosin-glucuronide, formononetin, calycosin-7-O-β-D-glucoside, and ononin were 0.524 mg/mL, 0.505 mg/mL, 0.509 mg/mL, 0.526 mg/mL, 0.512 mg/mL, and 0.508 mg/mL, respectively. The stock solution was diluted with phosphate-buffered saline (PBS) to prepare a series of standard solutions. All solutions were refrigerated at 4°C.

### 2.4 Preparation of specific inhibitor solutions

Specific inhibitor solutions with the following concentrations were prepared in methanol: 0.126 μg/mL α-naphthoflavone, 0.101 μg/mL ticlopidine, 0.104 μg/mL fluconazole, 0.106 μg/mL quinidine, 0.101 μg/mL clomethiazole, and 0.101 μg/mL ketoconazole. Before use, each inhibitor solution was diluted with PBS (1 mM, pH, 7.0) to prepare the required concentration and then stored at 4°C.

### 2.5 Preparation of mouse liver microsomes

Before the experiment, the mice were fasted for 12 h, and were euthanized after blood was taken from the eye sockets. The liver was quickly removed and perfused with pre-cooled saline through the hepatic portal vein until the liver was earthy yellow, washed three times with 1.15% KCl solution, wiped dry with filter paper, and then weighed. The liver was then cut up, homogenized with three times the volume of ethylene diamine tetraacetic acid (EDTA) in an ice bath, and centrifuged at 4°C for 10 min at 3,000 g. The supernatant was filtered, and the filtrate was centrifuged at 4°C for 20 min at 12,000 g. The supernatant was then centrifuged at 4°C for 45 min at 100,000 g, and the precipitate was resuspended in a resuspension solution (EDTA homogenate containing 20% glycerol). The protein concentration of the microsomes was determined using the BCA protein concentration determination kit, and the microsomes were aliquoted and pre-stored at −20°C for 60 min and transferred to −80°C for storage.

### 2.6 Liver microsomal incubation system and sample preparation

The total volume of the reaction was kept at 200 μL. The *in vitro* incubation system comprised of 50 μL liver microsomes (1 mg/mL), 50 μL specific inhibitor solution, 20 μL nicotinamide adenine dinucleotide phosphate (NADPH, 1 mmol/L), and 80 μL standard solution (100 ng/mL). The solution containing liver microsomes, specific inhibitors, and the standard solution was pre-incubated for 5 min at 37°C. NADPH, pre-incubated for 5 min at 37°C, was added to commence the reaction, and the solution was incubated for 60 min at 37°C on a thermostatic oscillator. At the end of the reaction, 400 L of ice-cold methanol containing the IS (the final concentrations of digoxin and puerarin were 0.745 μg/mL and 1 μg/mL, respectively) was added to terminate the reaction. The samples were vortexed for 1 min and centrifuged for 10 min (15,000 r/min). The contents of six active components in the supernatant were determined by HPLC-MS-MS. All reactions were performed in triplicate.

### 2.7 Preparation of quality control samples

The three concentrations of quality control samples were prepared by taking 50 μL of inactivated liver microsomes and adding 50 μL PBS solution, 80 μL mixed solution containing six components (astragaloside IV, glycyrrhizic acid, calycosin-glucuronide, formononetin, ononin, calycosin-7-O-β-D-glucoside) with low, medium, and high concentrations, 20 μL NADPH, 200 μL methanol, and 200 μL internal standard solution.

### 2.8 HPLC-MS/MS analysis

#### 2.8.1 Conditions of HPLC-MS/MS

An Acquity HPLC system (Shimadzu Corp., Kyoto, Japan) equipped with a Q-Trap^®^ 5,500 triple quadruple mass spectrometer (AB Sciex, Framingham, MA, United States) was utilized for HPLC-MS/MS. Multiple reaction monitoring (MRM) was employed to achieve mass spectrometric quantification. The parameters of the mass spectrometer were optimized as follows: source temperature at 600°C, nebulizer pressure at 55 psi, curtain gas at 30 psi, auxiliary gas at 55 psi, and ion spray voltage at −4.5 kV (−) or 5.5 kV (+). The mass spectrum parameters are shown in Table 2.

**TABLE 2:** Mass spectrum parameters of HPLC-MS/MS.

Compound	Q1	Q2	DP (Volts)	CE (Volts)	EP (Volts)	CXP (Volts)
	Mass	Mass				
Astragaloside IV	807.4	627.4	168	67	10	16
Glycyrrhizic acid	824.4	309.4	120	40	10	13
Calycosin-glucuronide	285.3	213.2	180	51	10	13
Formononetin	267.0	252.0	−108	−10	−10	−13
Calycosin-7-O-β-D-glucoside	447.1	285.2	109	29	10	13
Ononin	431.3	269.1	−120	−25	−10	−13
Digoxin	825.3	649.5	−108	−30	−10	−13
Puerarin	417.1	267.1	110	35	10	13

The liquid chromatography analyses of the six effective components were conducted using an ACEExcel2C18-AR column (100 × 2.1 mm, 2 μm, Advanced Chromatography Technologies Ltd., Aberdeen, United Kingdom) at 30°C. Analysis was completed with a gradient elution of 0.1% formic acid (A) and acetonitrile (B). The flow rate was 0.4 mL/min. The gradient elution procedure was as follows: 0–0.6 min at 10% B, 0.6–2 min at 10%–30% B, 2–6 min at 30%–65% B, 6–8 min at 65%–90% B, 8–9 min at 90%–90% B, 9–9.1 min at 90%–10% B, and 9.1–12 min at 10% B.

#### 2.8.2 Method validation

##### 2.8.2.1 Specificity

Briefly, 50 μL of inactivated liver microsomes were mixed with 50 μL of mix specific inhibitor, before adding 80 μL of PBS solution, 20 μL of NADPH, and 400 μL of methanol. Subsequently, the samples were vortexed for 1 min and sonicated for 3 min. After precipitating the protein, the samples were centrifuged at 15,000 r/min for 10 min, and the supernatant was collected to obtain sample A. Next, 50 μL of inactivated liver microsomes were mixed with 50 μL of mix specific inhibitor before we added 80 μL of the mixed solution of six components (astragaloside IV, glycyrrhizic acid, calycosin-glucuronide, formononetin, ononin, calycosin-7-O-β-D-glucoside) prepared in advance, 20 μL of NADPH, 200 μL of methanol, and 200 μL of internal standard solution. The remainder of the operations were the same as those outlined above for sample A, and the supernatant was collected to obtain sample B. Another sample containing six components was incubated according to the method described in section 2.6 to obtain sample C.

##### 2.8.2.2 Calibration curves and linearity

Briefly, 50 μL of inactivated liver microsomes were mixed with 50 μL of PBS solution, 20 μL of NADPH, 200 μL of methanol, 200 μL of internal standard solution, and then 80 μL of mixed standard solution of different concentrations was added to create a series of mixed working solutions. The samples were incubated according to the method described in section 2.6 and the supernatant was collected for determination using HPLC-MSMS. The calibration curves were drawn with the concentration (C) of each component as the horizontal coordinate X, and the ratio (A/A_i_) between the peak area of each component and internal standard as the vertical coordinate Y. Linear regression analysis was conducted using the weighted least squares method to obtain the regression equation. The lowest concentration in the calibration curve was used as the lower limit of quantification (LLOQ).

##### 2.8.2.3 Accuracy and precision

The low-, medium-, and high-concentration quality control samples were incubated according to the method described in Section 2.6. Accuracy and precision were assessed by repeated determination of six components in three concentration quality control samples six times a day for three consecutive days. The precision of the assay was computed using the relative standard deviation (RSD,%). The accuracy of the method was evaluated by the ratio of the measured concentration to the spiked concentration (%).

##### 2.8.2.4 Extraction recovery and matrix effects

To obtain sample A, the low*-*, medium*-,* and high*-*concentration quality control samples were incubated according to the method described in Section 2.6 and the supernatant was collected for determination by HPLC-MS/MS. Then, 50 μL of inactivated liver microsomes were treated according to the method described in Section 2.6, with the exception of adding the six components and internal standard. Subsequently, the corresponding low, medium, and high concentrations of the six components and internal standard mixed solution were added to the supernatant to obtain sample B. The low, medium, and high concentrations of the six-component standard solution (80 μL) were mixed with the internal standard (200 μL) and the initial mobile phase (320 μL) to obtain sample C. The ratio of the peak area of sample A to sample B was defined as the extraction recovery rate, while the ratio of the peak area of sample B to sample C was defined as the matrix effect.

##### 2.8.2.5 Stability

Low-, medium-, and high-concentrations of quality control samples were incubated according to the method described in Section 2.6. Subsequently, the quality control samples were stored at 25°C for 4 h, refrigerated for 48 h at −20°C, and underwent triple freeze-thaw cycles. The stability was evaluated by investigating the content changes of six components under different storage conditions.

### 2.9 Research on the dominant metabolic enzymes of six effective components of HQD

#### 2.9.1 Optimization of protein concentration in liver microsomal incubation system

The six-component mixed standard solutions were incubated with the liver microsomal sample of different protein concentrations (0, 0.25, 0.5, 0.75, 1, 2 mg/mL) for 60 min, each with six replicates. Subsequently, samples were prepared according to the methods described in Section 2.6. The residual contents of the six effective components in each group were determined by HPLC-MS/MS to calculate the metabolic conversion rate.

#### 2.9.2 Optimization of reaction time in liver microsomal incubation system

The mixed standard solution containing the six components was incubated with mouse liver microsomes at 37°C for different time (0, 15, 30, 45, 60, 90 min), each with six replicates. Subsequently, samples were prepared according to the methods described in Section 2.6. The residual contents of the six effective components in each group were determined by HPLC-MS/MS to calculate the metabolic conversion rate.

#### 2.9.3 Effect of each analyte’s concentration on its own metabolism

The standard solution of astragaloside IV, glycyrrhizic acid, calycosin-glucuronide, formononetin, calycosin-7-O-β-D-glucoside, and ononin was added to phosphate-buffered saline (PBS, pH 7.0) as desired for dilution to produce a series of mixed solutions (50, 100, 250, 500, 1,000 ng/mL). Each mixed solution sample was incubated with 1.0 mg/mL liver microsomes for 60 min. The residual contents of the six effective components were determined by HPLC-MS/MS to calculate the metabolic conversion rate.

#### 2.9.4 Effects of liver CYP enzymes specific inhibitors on the metabolism of six effective components

Α-naphthoflavone, quinidine, clomethiazole, ticlopidine, ketoconazole, and fluconazole were selected as specific inhibitors of CYP1A2, CYP2D22, CYP2E1, CYP2C37, CYP3A11, and CYP2C11, respectively (El-Sherbeni and El-Kadi, 2014; Xu et al., 2016). Forty-eight mice were randomly divided into eight groups of six mice each. The eight groups comprised six different specific inhibitor groups, a negative control group and a control group. For different specific inhibitor groups, mouse liver microsomes were incubated with related CYP450 enzyme specific inhibitors and mixed standard solutions. In the negative control group, mouse liver microsomes were incubated with mixed standard solution without specific inhibitors. In the control group, inactivated liver microsomes were combined with mixed standard solution without inhibitors and not incubated. After the samples were treated according to the methods described in Section 2.6, the residual level of the six effective components was determined for each group using HPLC-MS/MS. The residual contents of each component (%) = V_c_/V_0_*100 (where V_c_ is the residual amount of each group and V_0_ is the content of the control group).

### 2.10 Statistical analysis

All data were expressed as mean ± standard deviation. SPSS 23 was used for statistical analysis between the two groups. *p* ≤ 0.05 and *p* ≤ 0.01 were statistically significant between the two groups.

## 3 Results

### 3.1 Method validation of HPLC-MS/MS

#### 3.1.1 Specificity

**FIGURE 1:**
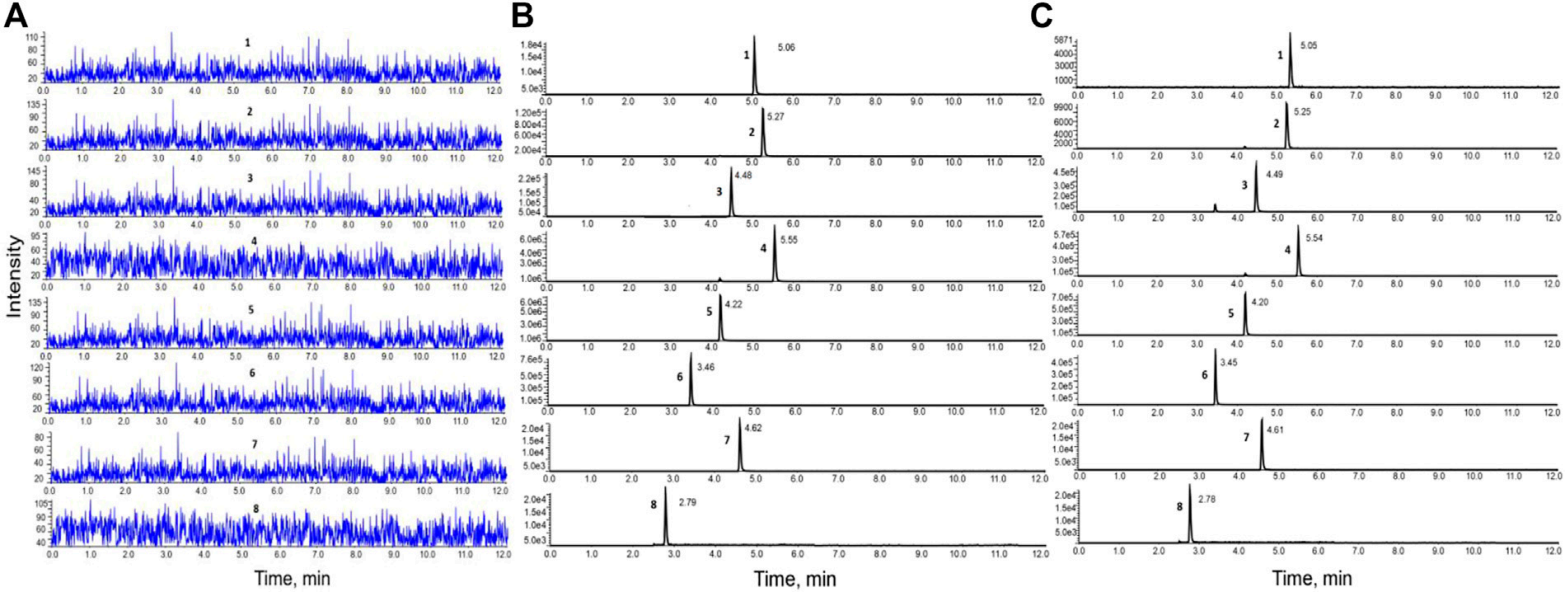
HPLC-MS/MS chromatograms of the six components. **(A)**. Blank inactivated liver microsomal sample; **(B)**. Blank inactivated liver microsomal sample mixed with the six components and internal standard; **(C)**. Samples obtained after incubation with liver microsomes.

The results demonstrated that the chromatographic peaks of the six components were well separated, with no interference between them. The retention times of astragaloside IV, glycyrrhizic acid, calycosin-glucuronide, formononetin, ononin, and calycosin-7-O-β-D-glucoside were 5.05 min, 5.25 min, 4.49 min, 5.54 min, 4.20 min, and 3.45 min, respectively (Figure 1).

#### 3.1.2 Calibration curves and linearity

Using the concentration of the six components as the abscissa (X) and the ratio of the peak area between each component and internal standard as the ordinate (Y), the weighted least squares method was employed for linear regression calculation to obtain the regression equation. All calibration curves displayed great linearity and coefficients of correlation ranging from 0.9992 to 0.9998. The regression equation and linear range for six components in the liver microsomal samples are listed in Table 3.

**TABLE 3:** Regression equation, linear range, and LLOQ for six components in liver microsomal samples.

Compound	Linear range (μg/mL)	Regression equation	*r*	LLOQ (μg/mL)
Astragaloside IV	0.01–5.34	*y* = 0.0012*x* − 0.0268	0.9994	0.01
Glycyrrhizic acid	0.01–5.09	*y* = 0.0014*x* + 0.031	0.9998	0.01
Calycosin-glucuronide	0.01–5.19	*y* = 0.0224*x* + 0.1288	0.9997	0.01
Formononetin	0.01–5.23	*y* = 0.0671*x* − 1.1249	0.9992	0.01
Ononin	0.01–5.07	*y* = 0.069*x* + 0.6873	0.9994	0.01
Calycosin-7-O-β-D-glucoside	0.01–5.05	*y* = 0.0349*x* + 0.394	0.9997	0.01

#### 3.1.3 Accuracy and precision

The inter- and intra-day precision ranged from 2.3% to 15.66% and 0.37%–17.43%, respectively. The inter- and intra-day precision and accuracy of six components in liver microsomal samples are summarized in Table 4. These results demonstrate that the method is accurate and reliable.

**TABLE 4:** Accuracy and precision of the six components in liver microsomal samples (x ± SD, *n* = 6).

Compound	Spiked conc. (ng/mL)	Accuracy (%)	RSD (%)	Inter-day precision RSD (%)	Intra-day precision RSD (%)
Astragaloside IV	20.12	111.2 ± 5.69	5.12	4.58	4.61
	108.6	103.5 ± 5.35	5.17	7.55	2.74
	4,012.7	100.9 ± 2.78	2.75	7.78	3.31
Glycyrrhizic acid	19.54	105.4 ± 9.72	9.22	2.30	17.43
	101.7	82.15 ± 1.34	1.63	5.78	1.63
	4,092.4	109.9 ± 8.54	7.77	7.58	7.77
Calycosin-glucuronide	19.60	90.77 ± 7.57	8.34	11.55	8.53
	105.2	88.60 ± 3.08	3.48	4.78	2.61
	4,065	103.3 ± 2.92	2.83	4.91	2.64
Formononetin	19.20	104.5 ± 2.38	2.28	9.29	4.40
	107.9	106.6 ± 2.26	2.12	15.66	2.12
	4,013.8	101.8 ± 13.93	13.68	8.52	13.68
Ononin	20.78	107.5 ± 7.3	6.80	9.16	0.37
	99.46	85.84 ± 3.76	4.38	8.29	4.96
	4,034	102.6 ± 3.99	3.89	3.98	3.95
Calycosin-7-O-β-D-glucoside	20.54	109.0 ± 7.64	7.01	7.36	5.20
	101.4	85.98 ± 2.23	2.60	6.17	2.60
	4,026	102.8 ± 3.43	3.34	2.98	3.34

#### 3.1.4 Extraction recovery and matrix effects

The mean matrix effect of the six components ranged from 90.57% ± 5.59% to 103.9% ± 6.33% with RSD <15%, and extraction recovery of the six components ranged from 88.06 ± 1.10 to 105.9 ± 10.46 with RSD <20%, which suggested that the recoveries of the six components were reliable in microsomal samples. Furthermore, there was no matrix interference. The matrix effect and extraction recovery are displayed in Table 5.

**TABLE 5:** Matrix effects and extraction recovery of the six components in liver microsomal samples (x ± SD, *n* = 6).

Compound	Matrix effect (%)	RSD (%)	Extraction recovery (%)	RSD (%)
Astragaloside IV	90.57 ± 5.59	6.17	88.06 ± 1.10	1.25
	103.9 ± 6.33	6.09	100.7 ± 6.80	6.75
	102.2 ± 1.57	1.54	105.7 ± 1.61	1.52
Glycyrrhizic acid	90.62 ± 7.30	8.06	95.58 ± 8.87	9.28
	92.31 ± 6.75	7.31	104.1 ± 18.25	17.52
	101.5 ± 4.92	4.85	105.9 ± 10.46	9.87
Calycosin-glucuronide	94.77 ± 0.69	0.73	92.61 ± 8.26	8.92
	95.67 ± 7.55	7.89	99.77 ± 9.31	9.33
	101.3 ± 1.84	1.82	105.7 ± 6.80	6.43
Formononetin	94.79 ± 9.21	9.72	97.5 ± 7.65	7.85
	85.27 ± 2.58	3.03	99.65 ± 8.13	8.16
	95.23 ± 8.13	8.54	104.5 ± 2.58	2.47
Ononin	90.65 ± 5.10	5.63	84.09 ± 0.89	1.06
	98.84 ± 4.22	4.27	101.0 ± 3.46	3.43
	102.2 ± 3.60	3.52	105.4 ± 5.98	5.67
Calycosin-7-O-β-D-glucoside	90.8 ± 9.70	10.68	90.58 ± 3.73	4.12
	98.25 ± 1.79	1.82	92.3 ± 9.04	9.79
	103.5 ± 2.11	2.04	105.9 ± 3.65	3.45

#### 3.1.5 Stability

**FIGURE 2:**
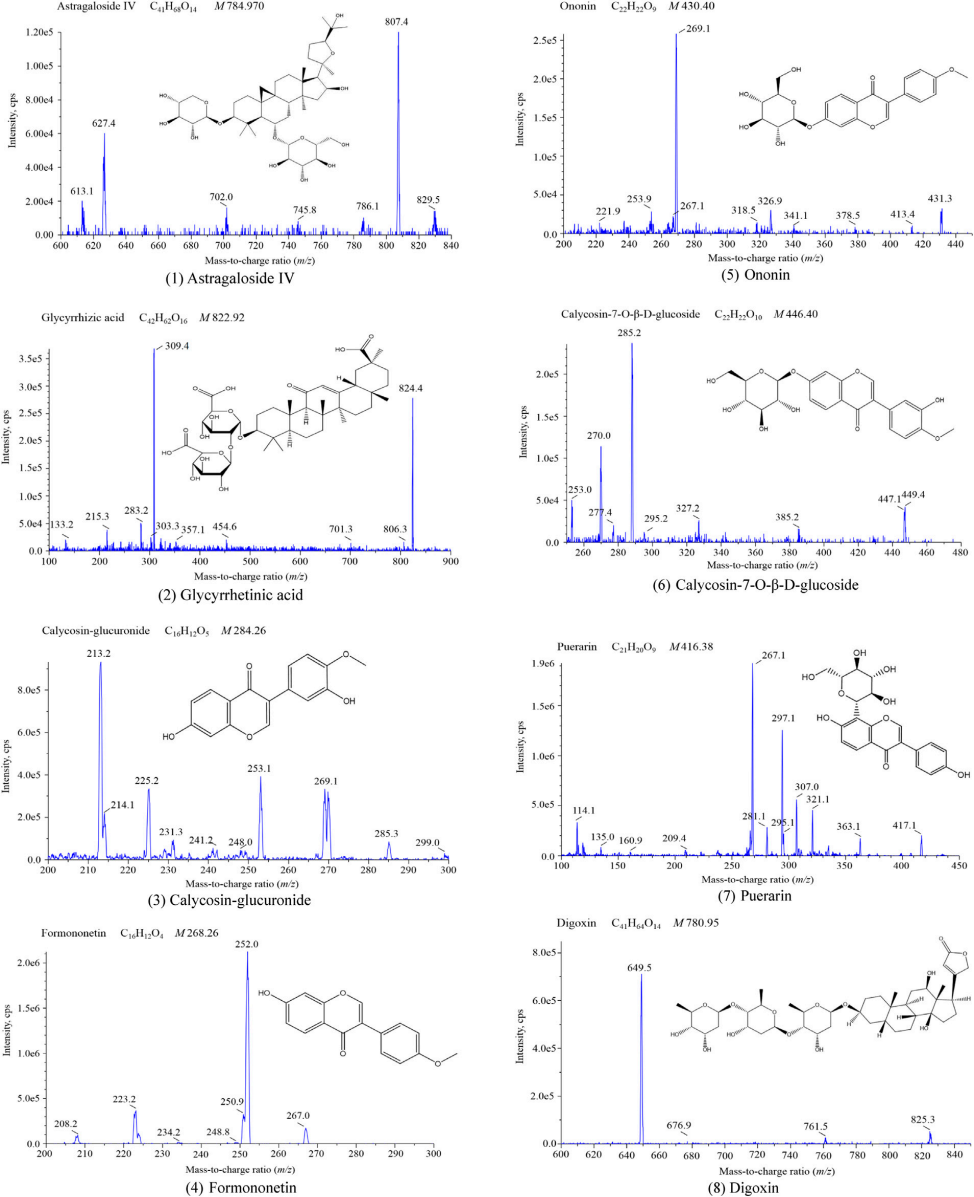
Mass spectrogram of the six components and internal standards (1) Astragaloside IV; (2) Glycyrrhetinic acid; (3) Calycosin-glucuronide; (4) Formononetin; (5) Ononin; (6) Calycosin-7-O-β-D-glucoside; (7) Puerarin; (8) Digoxin.

The result is displayed in Table 6. Our results revealed that the components are stable when stored at room temperature (approximately 25°C) for 4 h, refrigerated (−20°C) for 48 h and three freeze-thaw cycles. Therefore, the stability of samples conformed to the standard of biological sample determination. Figure 2.

**TABLE 6:** Stability of the six components in liver microsomal samples. (x ± SD, n = 6).

Compound	Spiked conc. (ng/mL)	25°C 4 h		−20°C 48 h		Three freeze–thaw cycles	
		Measured conc. (ng/mL)	RSD (%)	Measured conc. (ng/mL)	RSD (%)	Measured conc. (ng/mL)	RSD (%)
Astragaloside IV	20.12	21.46 ± 1.27	5.92	22.77 ± 2.56	11.24	21.13 ± 0.58	2.74
	108.5	109.3 ± 10.61	11.09	104.9 ± 3.58	3.41	98.03 ± 2.13	2.17
	4,012.8	4,034 ± 35.15	0.87	4,034 ± 72.45	1.80	3,902 ± 192.5	4.94
Glycyrrhizic acid	19.54	20.9 ± 2.31	11.05	20.52 ± 1.07	5.21	20.78 ± 2.95	14.19
	101.7	107.4 ± 6.62	17.93	97.94 ± 5.88	10.13	103.8 ± 1.54	17.38
	4,092	4,015 ± 219.5	5.47	4,042 ± 117.3	2.90	3,992 ± 183.4	4.59
Calycosin-glucuronide	19.60	20.59 ± 0.41	1.99	20.26 ± 0.39	1.92	20.85 ± 0.48	2.30
	105.2	97.47 ± 3	4.29	97.88 ± 1.01	1.03	99.14 ± 3.82	4.83
	4,065	4,111 ± 129.0	3.14	4,063 ± 135.3	3.33	4,104 ± 69.4	1.69
Formononetin	19.20	20.27 ± 0.11	0.54	21.10 ± 0.20	0.95	20.22 ± 0.09	0.45
	107.9	106.3 ± 0.21	1.18	107.0 ± 2.17	2.25	109.3 ± 1.78	0.94
	4,014	4,118 ± 428.3	10.40	4,033 ± 171.4	4.25	3,910 ± 110.6	2.83
Ononin	20.78	21.21 ± 1.43	6.74	20.29 ± 0.13	0.64	21.31 ± 0.32	1.50
	99.46	98.74 ± 2.44	2.47	98.51 ± 6.12	6.21	103.5 ± 2.26	2.18
	4,034	4,082 ± 121.9	2.99	4,034 ± 102.2	2.53	4,044 ± 162.4	4.02
Calycosin-7-O-β-D-glucoside	20.54	21.95 ± 1.71	7.79	20.53 ± 0.62	3.01	21.63 ± 1.13	5.11
	101.5	97.85 ± 6.11	11.09	97.13 ± 5.47	6.55	98.8 ± 2.02	9.74
	4,026	4,064 ± 84.17	2.07	4,132 ± 186.5	4.51	4,041 ± 131.2	3.25

### 3.2 Dominant metabolic enzymes of the six effective components in HQD

#### 3.2.1 Optimization of protein concentration in liver microsomal incubation system

**FIGURE 3:**
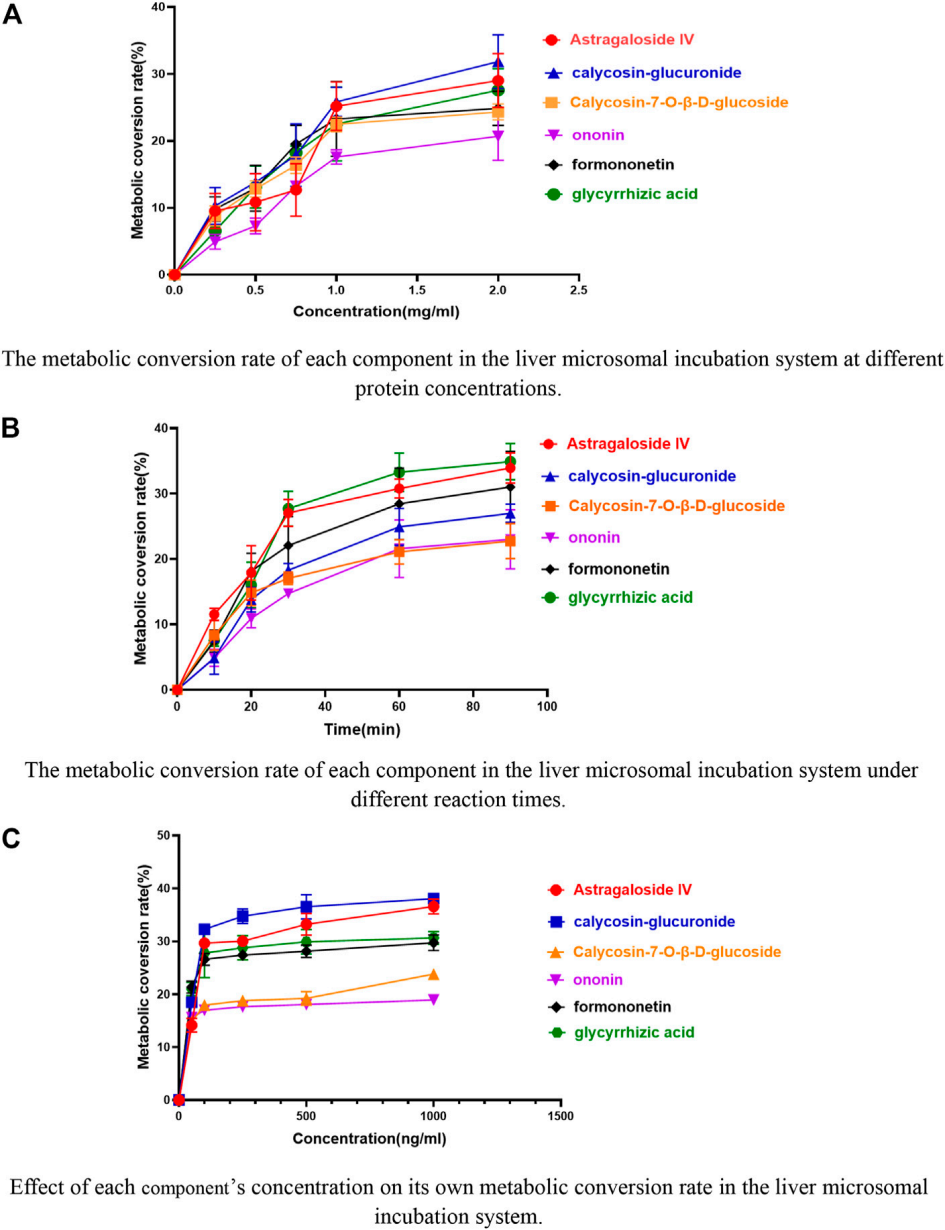
Effects of different conditions on the metabolic conversion rate of the six components in the liver microsomal incubation system. **(A)** The metabolic conversion rate of each component in the liver microsomal incubation system at different protein concentrations. **(B)** The metabolic conversion rate of each component in the liver microsomal incubation system under different reaction times. **(C)** Effect of each component’s concentration on its own metabolic conversion rate in the liver microsomal incubation system.

The results are shown in Figure 3. The metabolic conversion rate of astragaloside IV, glycyrrhizic acid, calycosin-glucuronide, formononetin, ononin, calycosin-7-O-β-D-glucoside initially increased with the increase in protein concentration. At higher concentration, the conversion rate for each analyte saturated. Therefore, for subsequent experiments, a 1 mg/mL concentration of liver microsomal protein was selected.

#### 3.2.2 Optimization of reaction time in liver microsomal incubation system

The metabolic conversion rate of astragaloside IV, glycyrrhizic acid, calycosin-glucuronide, formononetin, ononin, calycosin-7-O-β-D-glucoside increased with time, however, saturated after 60 min (Figure 3). Thus, 60 min was selected as the incubation reaction time.

#### 3.2.3 Effect of each analyte’s concentration on its own metabolism

The metabolic conversion rates of the six components increased with the increase of their respective concentrations and plateaued after increasing to 100 ng/mL (Figure 3). Therefore, 100 ng/mL was chosen as the concentration of the six components in the incubation system of liver microsomes.

### 3.3 Effects of specific liver CYP enzyme inhibitors on the metabolism of six effective components

**FIGURE 4:**
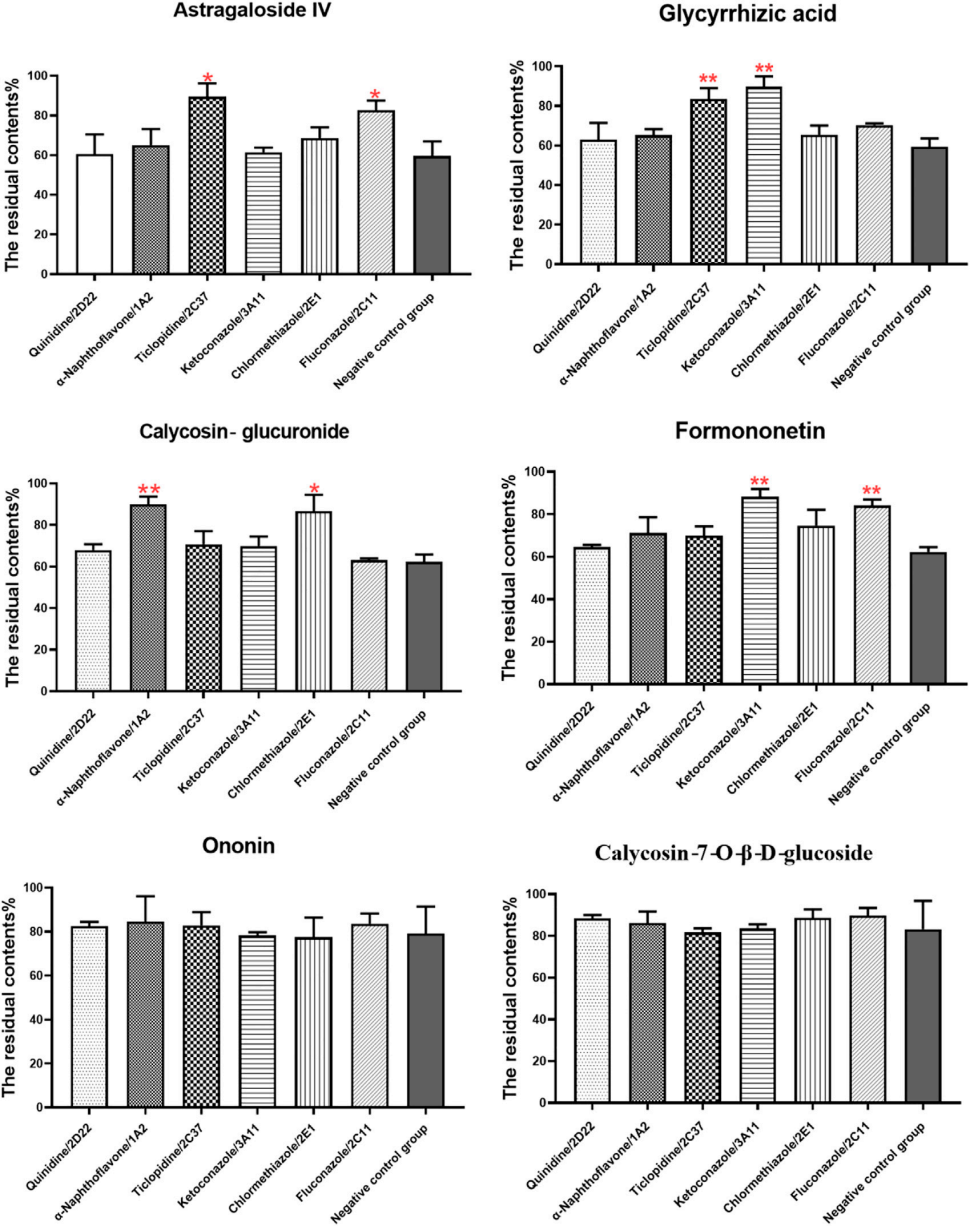
Effects of specific CYP enzyme inhibitors on the metabolism of the six components.

The effect of specific CYP enzyme inhibitors on the metabolism of six components was investigated (Figure 4). Compared to the control group, all components in negative control group were metabolized obviously, indicating that the condition of liver microsomal incubation system was reasonable and feasible. The addition of specific CYP enzymes inhibitors reveals that ticlopidine, a specific inhibitor of CYP2C37, prevented the metabolism of astragaloside IV and glycyrrhizic acid. Fluconazole, a specific inhibitor of CYP2C11, prevented the metabolism of astragaloside IV and formononetin. α-naphthoflavone, a specific inhibitor of CYP1A2, prevented the metabolism of calycosin-glucuronide. Clomethiazole, a specific inhibitor of CYP2E1, prevented the metabolism of calycosin-glucuronide. Lastly, ketoconazole, a specific inhibitor of CYP3A11, prevented the metabolism of formononetin and glycyrrhizic acid. However, the metabolism of calycosin-7-O-β-D-glucoside and ononin was not affected by the addition of these CYP enzymes inhibitors. Taken together, CYP2C37 and CYP2C11 may be involved in the metabolism of astragaloside IV, while the metabolism of calycosin-glucuronide may be related to CYP1A2 and CYP2E1. Moreover, CYP3A11 and CYP2C11 may be involved in the metabolism of formononetin, and the metabolism of glycyrrhizic acid may be related to CYP3A11 and CYP2C37.

## 4 Discussion

The db/db mouse model derived from the C57BL/6J mice has a defective leptin receptor gene with the characteristic of spontaneous diabetes mellitus 2 development. The pathogenesis of this model is very similar to that of human type 2 diabetes mellitus. After 4 weeks of age, this mouse strain gradually develops diabetic signs such as obesity, glycosuria, hyperglycemia, and hyperlipidemia, whereas complications of diabetic nephropathy occur at 8–12 weeks of age (Ponchiardi et al., 2013; Wang et al., 2022c). A previous study by our group found that blood glucose, triglycerides, cholesterol, blood creatinine, urea nitrogen, and 24-h urinary protein were significantly higher in 12-week-old db/db mice compared to control (*p* < 0.05). Simultaneously, our previous study found that many collagen fibers (blue) were visible in the glomerular basement membrane and tubular interstitium of 12-week-old db/db mice, and significantly more than that of the control, with obvious collagen fiber deposition and obvious glomerular and tubular lesions (Wang et al., 2022d). Therefore, 12-week-old db/db mice were used as the mouse model of diabetic nephropathy and db/m mice of the same age were used as the control. To avoid the effect of disease on drug metabolizing enzymes, the liver of the control mice was used to construct an *in vitro* liver microsome incubation system.

The CYP450 family of enzymes is the most important enzyme for the oxidative metabolism of drugs in the liver and an important object of preclinical drug metabolism studies. CYPs belong to phase I drug metabolizing enzymes, of which CYP2D6, CYP1A2, CYP2C19, CYP3A4, CYP2E1, and CYP2C9 are the six most dominant subtypes. These six subtypes account for approximately 80% of the liver’s total CYP450 enzymes, and 90% of drugs are metabolized by these six subtypes (Feng et al., 2019). Therefore, the six enzyme subtypes, CY2D6, CY1A2, CY2C19, CY3A4, CY2E1, and CY2C9, which have a large distribution and can metabolize numerous drugs, were selected for our investigation. Humans and mice are both mammals, and although many subtypes of CYP450 enzymes are unique to their species, they also have direct homology and similar functions. Therefore, it is possible to predict the physiological activity of different subtypes of CYP450 in humans by studying homologous subtypes in mice. Therefore, we chose to use mouse liver microsomes for metabolic studies (Martignoni et al., 2006; Li et al., 2017). Among them, mouse CYP1A2, CYP2D22, CYP3A11, CYP2E1, CYP2C11, and CYP2C37 are homologous to human CYP1A2, CYP2D6, CYP3A4, CYP2E1, CYP2C9, and CYP2C19, respectively. (Martignoni et al., 2006; Han et al., 2012; Yang et al., 2016; Weng et al., 2020). First, an *in vitro* incubation system for liver microsomes was established. According to the correspondence of CYP450 enzyme homologs between humans and mice, the related CYP450 enzyme-specific inhibitors quinidine (CYP2D22), α-naphthoflavone (CYP1A2), ticlopidine (CYP2C37), ketoconazole (CYP3A11), clomethiazole (CYP2E1) and fluconazole (CYP2C11) were added, and high-performance liquid chromatography-triple quadrupole tandem mass spectrometry (HPLC-MS/MS) was used to determine the metabolic residues of the six components (astragaloside IV, glycyrrhizic acid, calycosin-glucuronide, formononetin, ononin, calycosin-7-O-β-D-glucoside). However, the results also showed that CYP450 enzyme-specific inhibitors had no significant effect on calycosin-7-O-β-D-glucoside and ononin *in vitro.* Active ingredients bind, cleave, and metabolize intestinal flora after entering the intestine, which affects drug absorption and utilization, especially components containing glucosides such as calycosin-7-O-β-D-glucoside and ononin. Moreover, numerous drug transporters found on intestinal epithelial cells are targets for improving drug absorption and bioavailability, and calycosin-7-O- β-D-glucoside and ononin are likely metabolized by other metabolizing enzymes (Zhang et al., 2014; Wang et al., 2015).

## 5 Conclusion

An *in vitro* liver microsomal incubation method was adopted to study the effects of specific inhibitors of liver CYP450 enzymes on the metabolism of the six effective components so as to analyze the dominant CYP enzymes that may be involved in the metabolism of each component. Combined with the changes in enzyme activity in preliminary research, this study explored the possible reasons for the differences in pharmacokinetics of HQD under physiological and pathological states from the perspective of drug metabolism, as well as the possible drug-drug interactions caused by their clinical application. The results provide a theoretical basis for the clinical application and future development of traditional Chinese medicine.

## Data Availability

The original contributions presented in the study are included in the article/supplementary material, further inquiries can be directed to the corresponding author.
